# Future therapeutic options

**DOI:** 10.1186/1471-2474-14-S2-O7

**Published:** 2013-05-29

**Authors:** Arnold Reuser

**Affiliations:** 1 Department of Clinical Genetics, Center for Lysosomal and Metabolic Diseases, Erasmus MC University Medical Center, Rotterdam, The Netherlands

## 

In the year 500 before Christ, the Greek philosopher Heraclitus said, “if one does not expect the unexpected, one will not find it out, since the mind is not open to it, and one will not search for it.” This is, in short, the problem I have with predicting what the future therapeutic options for Pompe disease might be. Leaving out the unthinkable, there are still plenty of options left, but how realistic are they? Rather shortly after his discovery of the lysosomes, in 1955, Prof Christian De Duve hypothesized that lysosomal storage diseases could possibly be treated by enzyme therapy, the way we currently practise. Nevertheless, the first successful attempt at enzyme replacement therapy (ERT) in Gaucher disease, by Dr Roscoe Brady and colleagues, was not published until 1990. The trick they used to deliver human glucocerebrosidase to Kupffer cells in the liver and to macrophages in bone marrow and spleen was to modify the carbohydrates so the enzyme would be captured by the mannose-receptor on these cells. At present, enzyme replacement therapy is applied in Pompe disease, in Fabry disease, and in the mucopolysaccharidoses MPS I, II and VI. In all these latter diseases, the enzymes are targeted to the mannose 6-phosphate receptor. In my opinion ERT works best in Pompe disease, and its effect could be further improved by starting treatment with Myozyme or Lumizyme earlier and by using more than the prescribed dose (20 mg/kg), if deemed clinically necessary. Immunologic responses are contra-effective and need to be minimized. Increasing the mannose 6-phosphate content of rhGAA chemically, or by using an alternative production platform, is expected to improve the efficacy of ERT. Improving the binding of rhGAA to the mannose 6-phosphate / IGF-II like receptor by production of a fusion protein (GILT- technology) might equally lead to greater efficacy. The co-application of chaperones is seen as a further option. Meanwhile, gene therapy is considered the solution to all problems. The principle was demonstrated in 1971, but it was only three days ago that the first gene therapy was approved by regulatory authorities in the Western world. For the treatment of Pompe disease, gene therapy also holds great promise, but there might be a long way still to go. Interference with autophagy or exocytosis has been looming at the horizon for 50 years as a potential treatment for lysosomal storage disorders. This subject was rejuvenated, and present day insight offers an exciting window of yet unknown opportunities.

